# Plasmon-Sensitized Silica-Titanium Aerogels as Potential Photocatalysts for Organic Pollutants and Bacterial Strains

**DOI:** 10.1021/acsomega.3c04556

**Published:** 2023-09-04

**Authors:** Ecem Tiryaki, Ali Can Özarslan, Sevil Yücel, Miguel A. Correa-Duarte

**Affiliations:** † Nanomaterials for Biomedical Applications, Italian Institute of Technology (IIT), 16163, Genova, Italy; ‡ Department of Bioengineering, Faculty of Chemical and Metallurgical Engineering, Yildiz Technical University, 34220, Esenler, Istanbul, Turkey; § CINBIO, Universidade Vigo, 36310 Vigo, Spain; ∥ Southern Galicia Institute of Health Research (IISGS) and CIBERSAM, 36310, Vigo, Spain

## Abstract

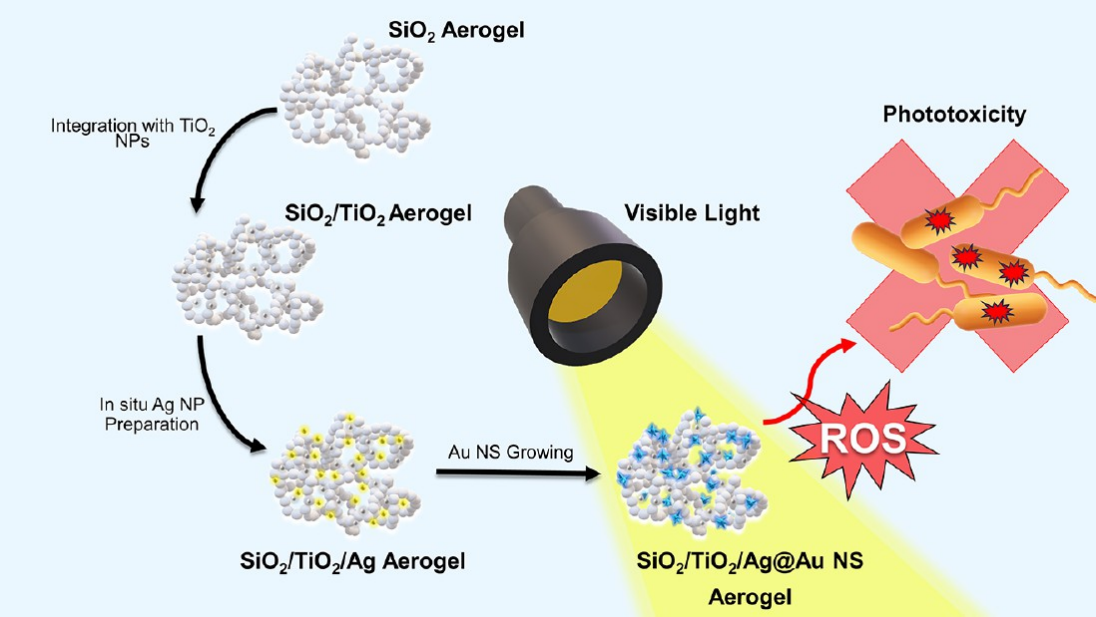

Photocatalysis reactions are of great interest as an effective tool against the profusely increasing population of antibiotic-resistant bacteria species. In particular, the promising evidence on plasmon-sensitized titanium dioxide (TiO_2_) photocatalysis inspired us to investigate their antibacterial activity stemming from the photogenerated reactive oxygen species (ROS). Herein, TiO_2_ nanostructures were grown *in situ* within a silica (SiO_2_) aerogel matrix with high surface area and porosity, and their ROS-related phototoxic effects against *Escherichia coli* bacteria were investigated under solar- and visible-light irradiations. Photodegradation profiles obtained from Rhodamine B (RhB) organic dye used as a chemical probe proved that the types of ROS produced by SiO_2_/TiO_2_ aerogels varied depending on the electromagnetic spectrum portion that was used during material irradiation. Further, the SiO_2_/TiO_2_ aerogel matrix was decorated with silver–gold nanostars (Ag@Au NSs) to enhance its photocatalytic efficiency under visible light irradiations. Our design showed that plasmon-enriched composite aerogels efficiently boosted ROS production under visible light exposures and that the structures containing Ag@Au NSs showed a much more effective antibacterial effect compared to their counterparts.

## 1 Introduction

Recently, there has been a huge upsurge in cases of bacterial infections caused by antibiotic-resistant bacteria that are highly aggressive to conventional treatment methods.^1^ As a result, there is a growing interest in developing new treatment approaches to combat antibiotic-resistant bacteria.^2^ Among them, photocatalysis, which involves the generation of reactive oxygen species (ROS) through the redox reactions arising from the interaction of photoactive materials and photon energy, has gained considerable attention.^1,3,4^ In terms of microbial disinfection, it has been already proven that the photogenerated ROS (such as hydroxyl radicals (^•^OH), superoxide anion radicals (^•^O_2_^–^), or hydrogen peroxide (H_2_O_2_)) induce bacterial cell death through oxidative stress, disrupting bacterial metabolism, and/or damaging protein/DNA structures.^5^

Titanium dioxide nanoparticles (TiO_2_ NPs) are efficient semiconductor materials widely employed in photocatalysis processes thanks to their low cost, excellent chemical stability, nontoxicity, and high photoactivity.^6^ Additionally, these materials exhibit intriguing properties as antibacterial photocatalysts by targeting bacterial cell structures through the production of ROS under UV light exposures. So far, different findings have been reported to elucidate the primary mechanism underlying the ROS-associated-phototoxicity of TiO_2_ NPs against numerous bacteria strains such as *Escherichia coli* and *Staphylococcus aureus* (*S. aureus*). For instance, Carré et al. (2014) evaluated the phototoxic effect of TiO_2_ NPs (Aeroxide P25) against *E. coli* bacteria and stated that ^•^O_2_^–^ radicals exhibited significant efficacy in oxidizing phospholipids and proteins within the bacterial cell membrane.^7^ Contrarily, in the other study, Yamaguchi et al. (2020) proposed that ^•^OH radicals, generated by TiO_2_ nanotube thin films, demonstrated potential bactericidal effects against both *E. coli* and *S. aureus* strains under UV light irradiation.^8^ Regarding the variable findings from different approaches, there is a need for further investigations into the toxicity of photogenerated ROS on bacteria strains. Besides, the limited absorption and utilization of photon energy in the visible and near-infrared (vis/NIR) regions restrict the potency of pure TiO_2_ NPs in real ecosystems.^9−11^ To address this limitation, researchers have developed methodologies to enhance the photoactivity of TiO_2_ NPs in the vis/NIR regions.^12^

One of the promising approaches is *in situ* growth of crystalline TiO_2_ NPs on thermally stable, porous, and high surface area materials since specific surface area and crystallinity play vital roles in photocatalysis processes. Particularly, silica (SiO_2_) aerogels are remarkable materials as functional supports thanks to their high specific surface areas and porosities, making them challenging in various applications, including drug delivery,^13,14^ catalysis,^15,16^ or antibacterial implementations.^17,18^ In the systems such as SiO_2_/TiO_2_ composite aerogels with improved properties, SiO_2_ aerogels act as excellent adsorbents by providing better adsorption centers and enriching organic/microbial entities into their pores, while TiO_2_ NPs serve as photocatalytically active sites for decomposing pre-enriched organic molecules.^19,20^ Moreover, the floating ability of low-density SiO_2_ aerogels allows them to be easily recovered from the environment after their reactions.^21^

In addition to improving the final structural properties of TiO_2_ NPs, amplifying their optical properties via plasmonic NPs is a considerable strategy to support their antibacterial effects under visible light.^9^ Plasmonic Ag NPs exhibit amplified electromagnetic responses due to their localized surface-plasmon resonance (LSPR) within the visible spectrum, rendering them promising photosensitizers in TiO_2_ NPs photocatalysis. Moreover, Ag NPs demonstrate proven antibacterial properties attributed to the release of Ag^+^ ions, resulting in a dual effect of enhancing the antimicrobial activities of TiO_2_ NPs.^22,23^ Nevertheless, Ag NPs are highly sensitive to oxidation, and the toxicity of Ag^+^ ions is comparatively lower than that of the generated ROS, contingent upon the concentration of metal ions released during the catalytic process.^23,24^ Hence, the development of hybrid Ag@Au metals as bimetallic materials with improved chemical stability and enhanced optical properties compared to their individual counterparts has gained prominence.^25,26^ Fabrication of these bimetallic structures in anisotropic morphology such as nanostars (NSs) characterized with intense electromagnetic hotspots at their tips further heightens the LSPR effect and thus overcomes disadvantages associated with Ag NPs in phototoxicity applications.^27,28^

**Scheme 1:**
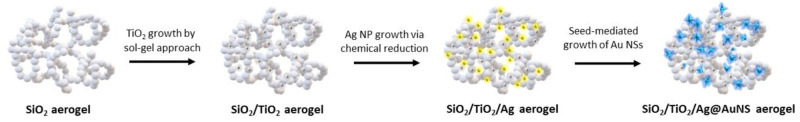
Schematic Illustration of Fabrication of Plasmon-Enriched SiO_2_/TiO_2_ Aerogels

Herein, the potential of SiO_2_/TiO_2_ composite aerogels decorated with plasmonic Ag@Au NSs to be effective antibacterial materials through their photocatalytic activities was investigated. For this, SiO_2_ aerogels were first synthesized by the sol–gel method and then used as a matrix for the growth of anatase TiO_2_ NPs. SiO_2_ aerogel served as a remarkable matrix that not only facilitated the adsorption of molecules from the environment but also ensured the homogeneous distribution of TiO_2_ NPs and plasmonic Ag@Au NSs growing *in situ* in their backbone. The plasmonic entities were fabricated on the SiO_2_/TiO_2_ aerogel matrix in two steps in which first Ag NPs were obtained via a chemical reduction process and then served as nucleation centers for further growth of Ag@Au NSs via seed mediated growth (Scheme 1). The photocatalytic efficiencies of composite aerogel systems, decorated with either Ag NPs or Ag@Au NSs, were examined under visible-light irradiation, assessing the photocatalytic decomposition of Rhodamine B (RhB) organic dye as a chemical probe and the phototoxicity against *E. coli* bacteria. Hence, by the design of a chemically stable and optically advanced system, it is aimed to reveal the synergistic antibacterial activity of plasmon-enriched SiO_2_/TiO_2_ aerogels associated with ROS production and toxic metal ion release.

## 2 Experimental Section

### 2.1 Materials

Sodium silicate (Na_2_O/3.2SiO_2_) was purchased from the Ege Kimya Co. Inc. (Turkey). Ethanol (≥99.9%), *n*-hexane (95%), and acetic acid (≥99.9%) were obtained from Merck (Germany). Titanium(IV) butoxide (97%, TNBT), sodium chloride (≥99.5%, NaCl), Rhodamine B (≥95% RhB), hexadecyltrimethylammonium bromide (≥99%, CTAB), silver nitrate (99%, AgNO_3_), hydrochloric acid (37%, HCl), l-ascorbic acid (%99, L-AA), sodium citrate tribasic dihydrate (≥98%, CA), terephthalic acid (98%, TA), tetrachloroauric acid (99.9%, HAuCl_4_·3H_2_O), and Ampicillin (Amp) were purchased from Sigma-Aldrich. TiO_2_ NPs (99%, anatase, 5 nm size) were purchased from Nanostructured & Amorphous Materials, Inc. (Katy, TX, USA). Yeast extract and tryptone were obtained from Condalab. Milli-Q water with a resistivity higher than 18.2 MΩ cm^–1^ was used for all of the preparations.

### 2.2 Method

#### 2.2.1 Synthesis of Silica Aerogels

Sodium silicate-based silica aerogels were synthesized according to our previous report.^13^ Briefly, the formation of the gel was achieved by maintaining a diluted Na_2_O/3.2SiO_2_ solution at pH 4 with the help of HCl. The resulting gel was aged for 24 h at 50 °C and then washed several times with water and ethanol to remove the residual salts and water in the gel backbone, respectively. Following, the aging and solvent-exchange processes at 50 °C for 24 h were repeated in ethanol and *n*-hexane. Finally, SiO_2_ aerogels were obtained by drying the wet gel in a spray dryer (BUCHI Mini Spray Dryer B-290) with an inlet temperature of 190 °C and an outlet temperature of 80 °C.

#### 2.2.2 Synthesis of SiO_2_/TiO_2_ Composite Aerogels

SiO_2_/TiO_2_ composite aerogels were synthesized according to the previously reported method with some modifications.^29^ Briefly, 0.5 g of SiO_2_ aerogels and 0.6 g of TNBT were dispersed in 20 mL of ethanol, and then 0.8 mL of acetic acid was added dropwise to initiate the hydrolysis reaction. The resulting colloidal solution was stirred continuously for another hour, then aged at room temperature for 48 h. The colloid was dried in an oven at 80 °C before being calcined at 600 °C for 2 h.

#### 2.2.3 Synthesis of SiO_2_/TiO_2_/Ag NPs Composite Aerogels

Ag NPs were synthesized *in situ* on the as-synthesized SiO_2_/TiO_2_ aerogels. Nucleation and growth processes of Ag NPs were achieved by following the previously reported chemical reduction method.^30^ Thus, first, an aqueous solution of CA (34 mM), AgNO_3_ (58.8 mM), and NaCl (20 mM) was prepared with 2.5 mL of final volume and stirred for 5 min at room temperature. Separately, 0.08 mL of L-AA aqueous solution (0.1 M) was added to the SiO_2_/TiO_2_ aerogel (0.5 g) dispersion in 25 mL of boiled water. After a minute, the premixture was added into the SiO_2_/TiO_2_ aerogel dispersion and stirred for 1 h. Finally, the dispersion was cooled and the obtained structures were washed by centrifugation at 4000 rpm for 30 min. To evaluate the effect of the Ti:Ag molar ratio on the photocatalytic activity of the catalyst, different concentrations of AgNO_3_ solution were added into the SiO_2_/TiO_2_ matrix.

#### 2.2.4 Synthesis of SiO_2_/TiO_2_/Ag@Au NSs Composite Aerogels

Ag@Au NSs were growth on SiO_2_/TiO_2_/Ag NPs via a seed-mediated growth approach based on the previous publication.^31^ Thus, AgNO_3_ at a final concentration of 5 × 10^–2^ mM was added to 20 mL of CTAB aqueous solution (50 mM). After 1 min of magnetic stirring, 0.25 mM HAuCl_4_·3H_2_O and 80 mM L-AA were added to the solution sequentially at 1 min intervals. Finally, 2 mL of SiO_2_/TiO_2_/Ag NPs composite (5 mg mL^–1^) was added, and the solution was stirred for 1 h. The obtained SiO_2_/TiO_2_/Ag@Au NSs structures were collected by centrifugation (4000 rpm, 30 min) and washed several times with Milli-Q water.

#### 2.2.5 Photocatalytic Activity Studies

The photocatalytic activities of the samples were assessed through the degradation of RhB organic dye in an aqueous solution under light irradiation using a 300 W xenon lamp. Thus, 5 mg samples were dispersed in 20 mL of RhB dye aqueous solution (10^–5^ M) and stirred overnight in the dark to ensure proper blending and to establish adsorption–desorption equilibrium before initiating the light exposure. During the light irradiation processes, the suspension was magnetically stirred, and the temperature of solution was maintained constant at 25 °C. 1.5 mL aliquots were taken from the sample at 30 min intervals to monitor changes in RhB dye absorbance values by using a UV–vis Spectrophotometer.

#### 2.2.6 Phototoxicity Assay against *E. coli*

The experiments for the evaluation of ROS-related phototoxicity were performed by using MG1655 *E. coli* bacteria transformed with the pGEN222 Omp-C-GFP plasmid^32^ as a model bacteria strain. First, *E. coli* bacteria was inoculated overnight at 37 °C with 220 rpm of agitation in 5 mL of Luria–Bertani (LB) broth which includes Amp (100 μg mL^–1^). Next, the bacterial culture was diluted to an optical density (OD) of 1, and 10^7^ colony-forming units (CFUs) from the bacterial culture were added to the aerogel dispersion, which contained 0.2 mL of sample (1 mg mL^–1^) and 1.7 mL of physiological water (PSW, with 0.9% of NaCl). Two sterilized vials were used for 3 h of experiments: one for irradiating with the 300 W Xe lamp; and the other to keep it in darkness with aluminum foil, as a control. After treatment, the bacteria for each sample were spread on LB-Amp agar plates and incubated at 37 °C for 18 h. The CFU count method was used to calculate estimated bacterial cell viability. Each experiment was performed in triplicate, and the results were reported as mean values.

### 2.3 Characterization

Transmission electron microscopy (TEM) analyses were performed by using a JEOL JEM1010 microscope (Tokyo, Japan) operating at an acceleration voltage of 100 kV. TEM samples were prepared by dropping 10 μL of NPs dispersion onto a 400 mesh Cu grid covered with a carbon film and drying at room temperature. Scanning electron microscopy (SEM) micrographs were obtained by conducting a FEI Quanta 200 Scanning Electron Microscope at 5.0 kV. High-resolution TEM (HRTEM) images and Energy Dispersive X-ray Spectroscopy in Scanning Transmission Electron Microscopy mode (STEM–EDS) analysis were performed in a JEOL JEM-2010F transmission electron microscope operating at an acceleration voltage of 200 kV. Nitrogen sorption at 77 K was performed in a Micromeritics 3Flex instrument. Before the measurements, the aerogel samples were degassed at room temperature for 12 h at 120 °C under a pressure of 0.1 Pa. The BET processing was carried out in the relative pressure range of 0.0–1.0. ζ-potential measurements were performed by using a Malvern Zetasizer Nano series. UV–visible-near-infrared (UV–vis-NIR) spectra were obtained by using a Hewlett-Packard HP8453 spectrophotometer (CA, USA). Diffuse reflectance UV–vis absorption spectra (UV–vis DRS) were obtained on a UV-2400 spectrophotometer (Shimadzu, Japan), and the band gap energy (*E*_g_) was calculated according to the Kubelka–Munk function. The crystallinity and crystalline phase of samples were characterized by performing X-ray diffraction (XRD) analysis with a Siemens D5000 diffractometer. Diffraction patterns were collected from 10° to 100° 2θ with scan parameters of 0.02° steps and 4 s counting time per step. The elemental analysis of prepared samples was performed by Inductively Coupled Plasma Optical Emission spectroscopy (ICP-OES). For this, aqueous solutions of samples were sonicated inside HNO_3_, HCl, and HF solution for 30 min in an ultrasonic bath and heated at 80 °C overnight. X-ray photoelectron spectroscopy (XPS) was performed by using a Thermo Scientific NEXSA instrument equipped with Al Kα monochromatized radiation at a 1486.6 eV X-ray source. Photoelectrons were collected from a takeoff angle of 90° relative to the sample surface. The measurement was done in Constant Analyzer Energy mode (CAE) with a 100 eV pass energy for survey spectra and a 20 eV pass energy for high-resolution spectra. Surface elemental composition was determined by using the standard Scofield photoemission cross sections. The atomic concentrations were determined from the XPS peak areas using the Shirley background subtraction technique and Scofield sensitivity factors. High-resolution XPS data were fitted with Gaussian–Lorentz functions after background subtraction using Avantage data processing software under the constraint binding energy (BE) shift and full width at half-maximum range (fwhm). Fluorescence microscope images were acquired using a Nikon Ti–U inverted microscope with a 10× objective. Images were captured by using the emission filters of fluorescein isothiocyanate (FTIC, excitation: 475/35 nm; emission: 530/43 nm; dichroic: 499) for GFP channels. Image acquisition was performed by using the NIS software.

## 3 Results and Discussion

### 3.1 Fabrication and Characterization of Composite Aerogels

**Figure 1:**
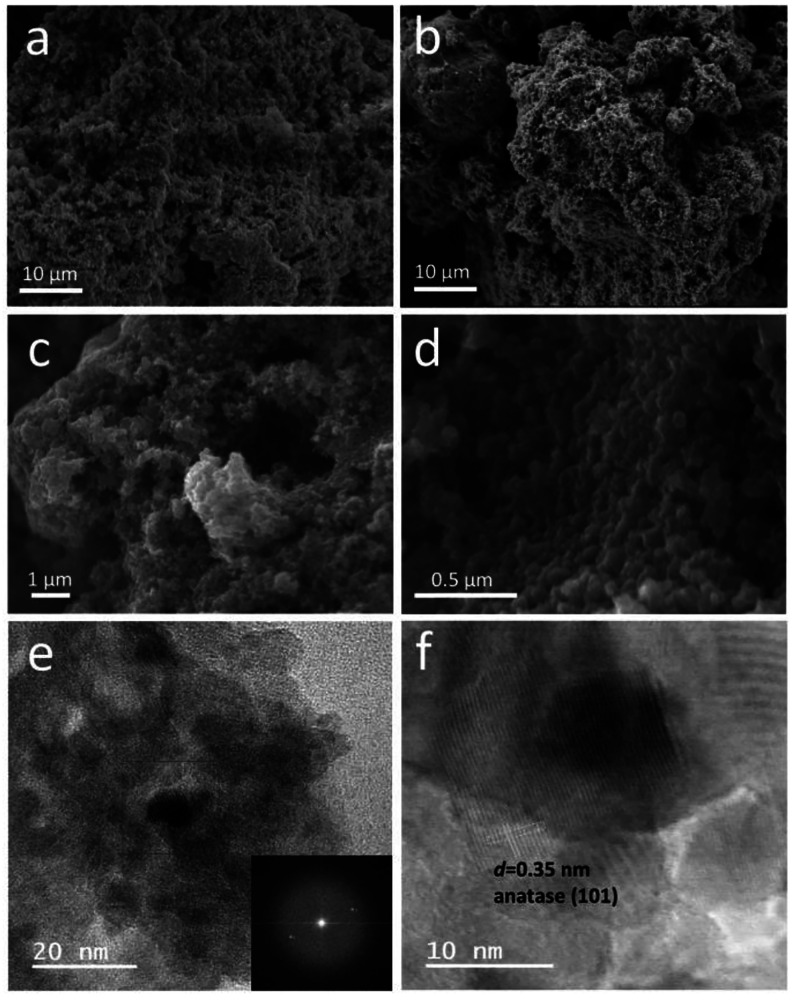
SEM micrographs of SiO_2_ aerogel (a,b), SiO_2_/TiO_2_ aerogels (c,d), and HRTEM images of SiO_2_/TiO_2_ aerogels (e,f).

To advance the photocatalytic activity and antibacterial performance of TiO_2_ NPs, we fabricated SiO_2_/TiO_2_ composite aerogels decorated with plasmonic metal nanostructures. Initially, SiO_2_ aerogel, serving as a mesoporous matrix for incorporating various metal components, was synthesized through the sol–gel approach using Na_2_O/3.2SiO_2_ as a silica precursor. Figure 1a,b presents SEM micrographs of the obtained silica aerogels, which were characterized by their grape-like morphologies organized by the small silica NPs to give a final highly porous aerogel matrix. Subsequently, crystalline TiO_2_ NPs were grown *in situ* within the SiO_2_ aerogel matrix to attain a composite aerogel structure. Thus, the sol–gel process was exerted to govern the hydrolysis and condensation of the TiO_2_ precursor, TNBT. At this stage, different concentrations of TNBT (0.04, 0.09, and 0.17 M) were incorporated into SiO_2_ aerogel matrix to investigate the effect of titanium amount on the final structural properties of the composite aerogel. Following the gel formation, the resulting structure was calcined to ensure the presence of anatase TiO_2_ on the silica aerogel backbone. SEM micrographs of the obtained SiO_2_/TiO_2_ composite aerogels, depicted in Figure 1c,d, exhibit morphological similarity to pure SiO_2_ aerogel structures by containing small grains and interconnected networks composed of irregular spherical NPs. The successful formation of the highly porous composite aerogel matrix was evident from the SEM images.

As previously reported, TiO_2_ aerogels synthesized as catalysts exhibit lower surface areas compared with SiO_2_ aerogels. A critical drawback of these structures is their lack of thermal stability, which leads to the collapse of their pores during calcination at elevated temperatures, resulting in a reduction in the final porosity and surface area.^33−35^ Contrariwise, in this study, a highly efficient composite aerogel structure was successfully attained by growing anatase TiO_2_ with homogeneous distributions within a thermally stable SiO_2_ aerogel matrix. Figure 1e,f represents HRTEM images of the obtained SiO_2_/TiO_2_ composite aerogels. Given that, SiO_2_/TiO_2_ composite has a crystal lattice as a result of the formation of anatase TiO_2_ after calcination at 600 °C. The interlayer distance was measured at 0.35 nm which confirmed the crystalline phase of anatase titanium (101).^36^ Similarly, XRD analysis confirmed the obtained anatase phase of TiO_2_ (Figure 3e). Accordingly, the XRD pattern indicated that SiO_2_ (black colored line) was present in the amorphous form, as characterized by a broad reflection at 2θ values of 22°,^29,37^ whereas SiO_2_/TiO_2_ composite aerogels (red colored line) exhibit diffraction peaks at 25°, 38°, 48°, 54°, 55°, and 63° that corresponds to (101), (004), (200), (105), (211), and (204) crystallographic planes of anatase titanium, respectively (JCPDS file Card no. 21–1272). From the pattern, it can be seen that only the anatase phase was present, while the rutile phase did not appear. Anatase TiO_2_ is an n-type semiconductor that is often preferred in photocatalysis applications, playing a crucial role in electron conductivity.^38^ The transition of titanium from the amorphous phase to the anatase phase occurs during calcination at temperatures higher than 400 °C, while the transition to the rutile phase is observed at higher temperatures.^29^ However, the enhancement of the titanium tolerance to the elevated temperatures and thereby the preservation of the anatase phase as a dominant phase can be achieved by dispersing titanium in a thermally stable SiO_2_ aerogel matrix.^29^ Accordingly, Ji et al., (2021) observed that as the calcination temperature increased by 800 °C, the mixture of rutile/anatase phase appeared in TiO_2_ NPs which embedded in SiO_2_ aerogels and consequently provided better photoactivity. However, as a disadvantage of the calcination process at increasing temperatures, SiO_2_ aerogel structure deteriorates, which significantly reduces its adsorption capacities.^36^ To avoid possible pore collapses and damages in SiO_2_/TiO_2_ aerogel backbone, we performed the calcination at 600 °C for 2 h so that the composite matrix may adsorb the organic molecules before and after their photodegradation.

**Figure 2:**
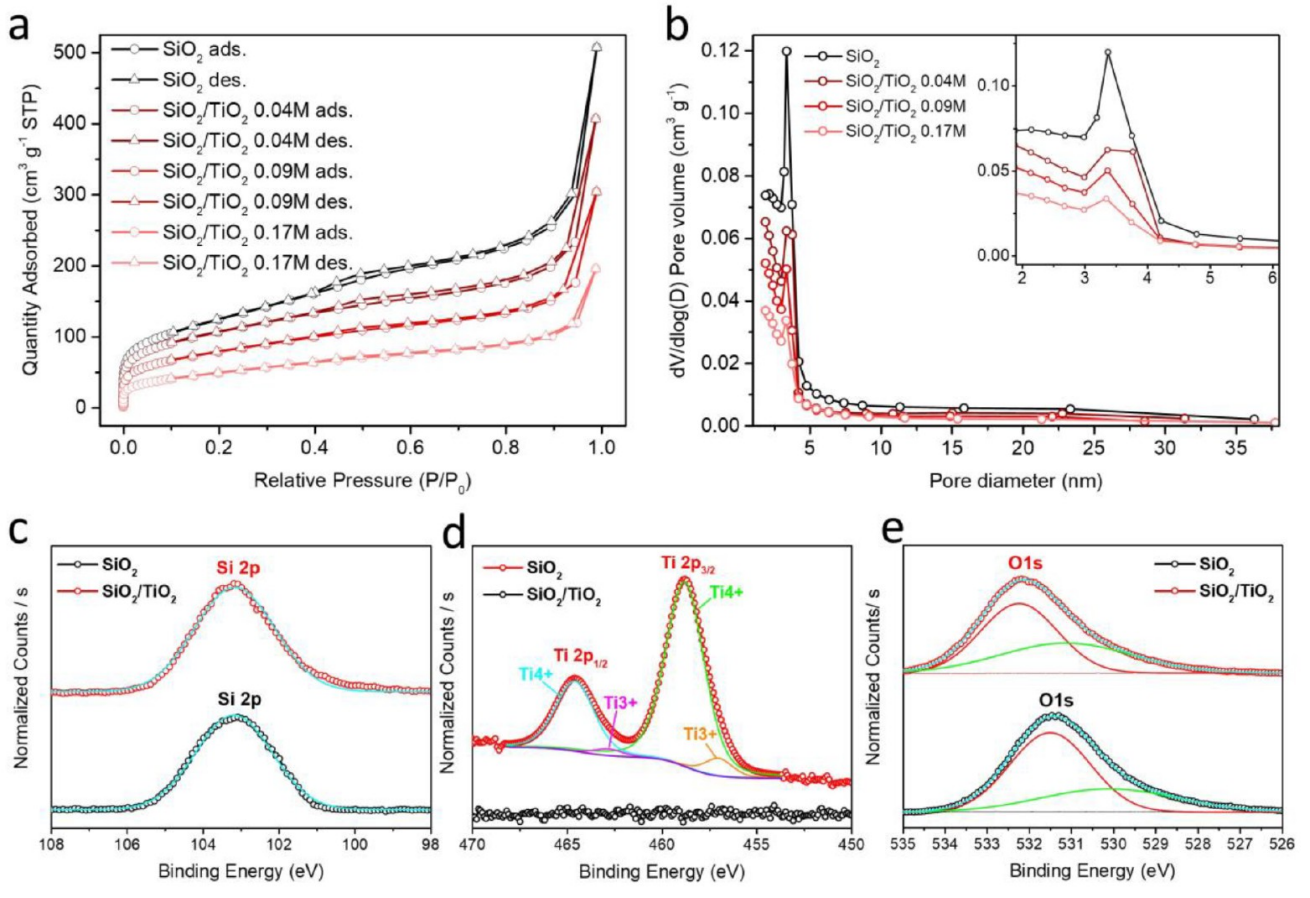
N_2_ adsorption/desorption isotherms (a) and pore size distributions (b) of SiO_2_ aerogels and SiO_2_/TiO_2_ composite aerogels with different concentrations of titanium precursor (0.04, 0.09, and 0.17 M), XPS spectra (fitted by Gaussian) of SiO_2_ and SiO_2_/TiO_2__0.09 M aerogels for the comparison of the states of Si 2p, Ti 2p, and O 1s (c–e).

The nitrogen adsorption/desorption isotherm analysis was performed to characterize the surface area by the Brunauer–Emmett–Teller (BET) method and pore size distribution and pore volume values by the Barrett–Joyner–Halenda (BJH) method. As shown in Figure 2a,b, SiO_2_ and SiO_2_/TiO_2_ aerogels exhibited type IV isotherms and H3 hysteresis at *P*/*P*_0_ = 0.25 according to the IUPAC classification. The specific surface area and pore volume/size values of SiO_2_ and SiO_2_/TiO_2_ aerogels with different titanium concentrations are presented in Table S1. The results showed that pure SiO_2_ aerogels were obtained with 447.9 m^2^ g^–1^ of specific surface area and 0.73 cm^3^ g^–1^ and 8.1 nm of pore volume and average pore size, respectively. Unavoidably, the surface areas and pore volumes of the final composite aerogel decreased as the titanium concentration increased. Accordingly, we decided to perform photodegradation and phototoxicity studies using SiO_2_/TiO_2__0.09 M samples in order to ensure maximum adsorption of organic/microbial pollutants and thus efficient photocatalytic activity.

Finally, XPS analysis was performed for elemental analysis, detection of the chemical environment, and the oxidation states of elements present at the sample surfaces. Figure 2c–e and Figure S1 present the XPS survey spectra of SiO_2_ aerogel and SiO_2_/TiO_2_ composite aerogels which confirmed the main elements as Si, O, and Ti on the surface of aerogel composite structures, suggesting the successful compositing between SiO_2_ and TiO_2_. The binding energy of Si 2p_1/2_ in the sample SiO_2_/TiO_2_ aerogels was detected at 103.1 eV which is lower than that of pure SiO_2_ (103.7 eV). This result may indicate that the oxidation state of Si has been altered as a result of combining with TiO_2_. According to the Ti 2p signals of the SiO_2_/TiO_2_ sample, Ti 2p_1/2_ and Ti 2p_3/2_ with the main titanium species Ti^4+^, are located at 464.6 and 458.8 eV, respectively, indicating the existence of Ti(IV) in TiO_2_ structures. Moreover, additional minor contributions from another species with lower oxidation numbers were also observed. The binding energies of these minority species (462.8 and 457 eV for Ti 2p_1/2_ and Ti 2p_3/2_, respectively) matched well with Ti^3+^ resulting from the defects in the lattice of structure during the synthesis.^39^ Finally, the O 1s signals of the samples are shown in Figure 2e which are located at 531.4 and 532.1 eV for SiO_2_ and SiO_2_/TiO_2_ aerogels, respectively. In the case of SiO_2_/TiO_2_, the two peaks at 530.8 and 532.2 eV in the O 1s high-resolution spectra reveal the presence of lattice O and Ti–O–Si, respectively.^36^Table S2 summarizes all the binding energies of elements detected on the surfaces of samples and their atomic percentages. SiO_2_ aerogels were obtained with 1:2 stoichiometry of Si and O elements (Si: 32.48%, O: 64.86%). Accordingly, the proportion of silicon decreased as a result of the additional contributions of TiO_2_. Moreover, ICP-OES analysis confirmed the similar elemental distribution of the obtained composite aerogel in which silicon was the primary constituent (Table S3). This outcome arises from the deliberate effort to maintain relatively low TiO_2_ percentages within the SiO_2_ aerogel matrix during the in situ growth phase of TiO_2_. This approach was employed to prevent the potential aggregation of NPs, which could otherwise result in reduced surface area and thereby diminished photocatalytic efficiency.

**Figure 3:**
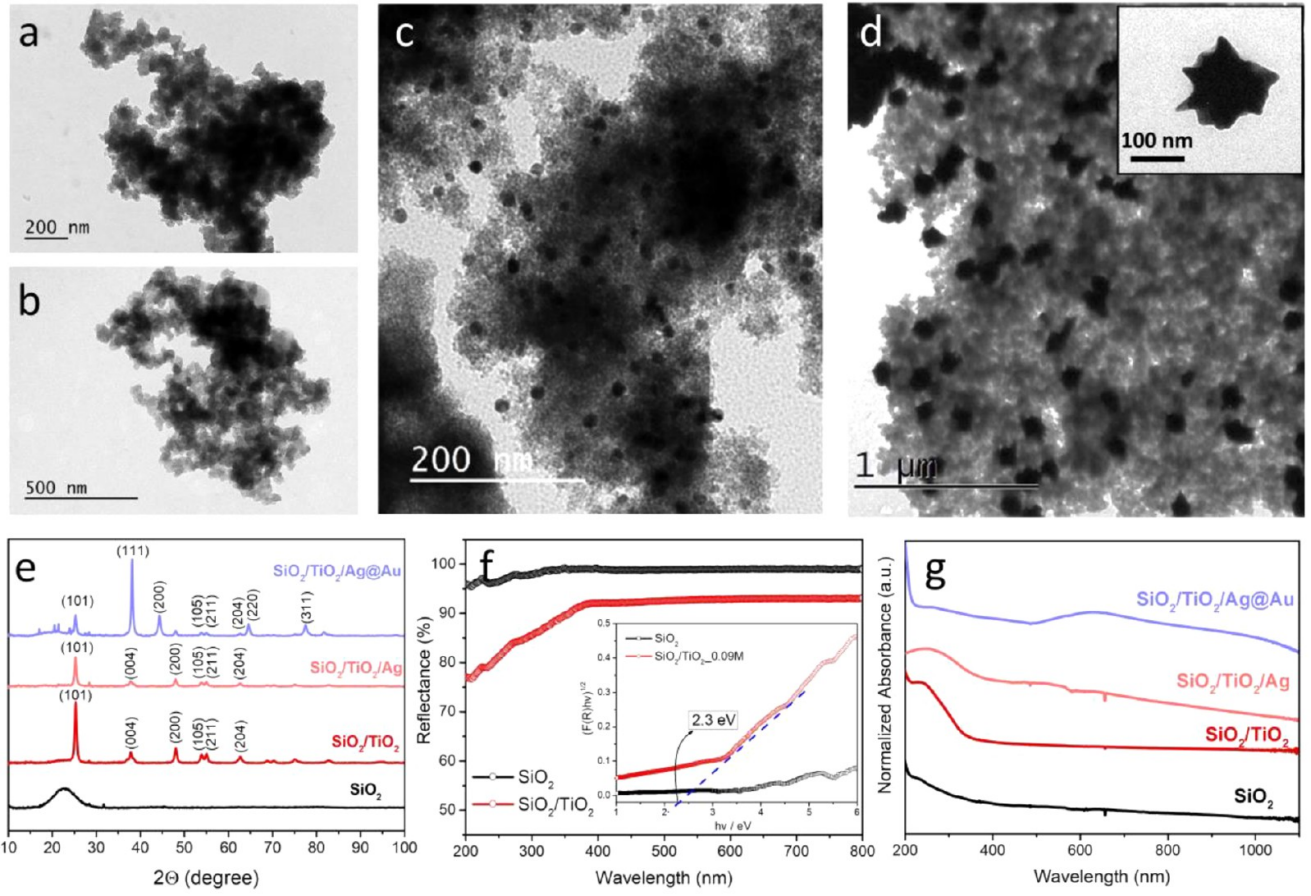
TEM images of SiO_2_ aerogel (a), SiO_2_/TiO_2_ aerogels (b), SiO_2_/TiO_2_/Ag NPs (c), and SiO_2_/TiO_2_/Ag@Au NSs (Ag@Au NSs alone, inset) (d), XRD pattern of composite aerogels (e), diffuse reflectance spectra of SiO_2_ and SiO_2_/TiO_2_ aerogels, and Tac plots of samples for their band gap calculations using the Kubelka–Munk function for indirect semiconductor (f), and UV–vis spectra of obtained composites (g).

Following, plasmonic NPs were grown *in situ* on SiO_2_/TiO_2_ composite aerogels to improve the optical properties of the composite and thus enhance their photocatalytic and antibacterial activities through plasmon-induced mechanisms. In this regard, seed-mediated growth approach was carried out using as-synthesized Ag NPs as nucleation centers for the anisotropic growth of Ag@Au NSs. For this, first, Ag NPs were synthesized on SiO_2_/TiO_2_ aerogel matrix by chemical reduction technique (Figure S2a, schematic representation). The principle of this method involves the adsorption of silver precursor on the aerogel surface and the chemical reduction of dissolved Ag^+^ ions in the presence of reducing agents. Herein, Ag^+^ ions were reduced and nucleated in the presence of an L-AA reducing agent and CA stabilizer. Finally, Ag NPs with spherical morphologies occurred through the further growth and aggregation of Ag nuclei by the Ostwald ripening phenomenon. Figure 3a,b presents TEM images of SiO_2_ and SiO_2_/TiO_2_ aerogels with small NP network structures giving a final porous morphology. Following their growth on the aerogel matrix, Ag NPs appeared in spherical morphology with an average size of 34.7*/1.02 nm indicating successful growth and homogeneous distribution on the composite aerogels (Figures 3c and S3b).

**Figure 4:**
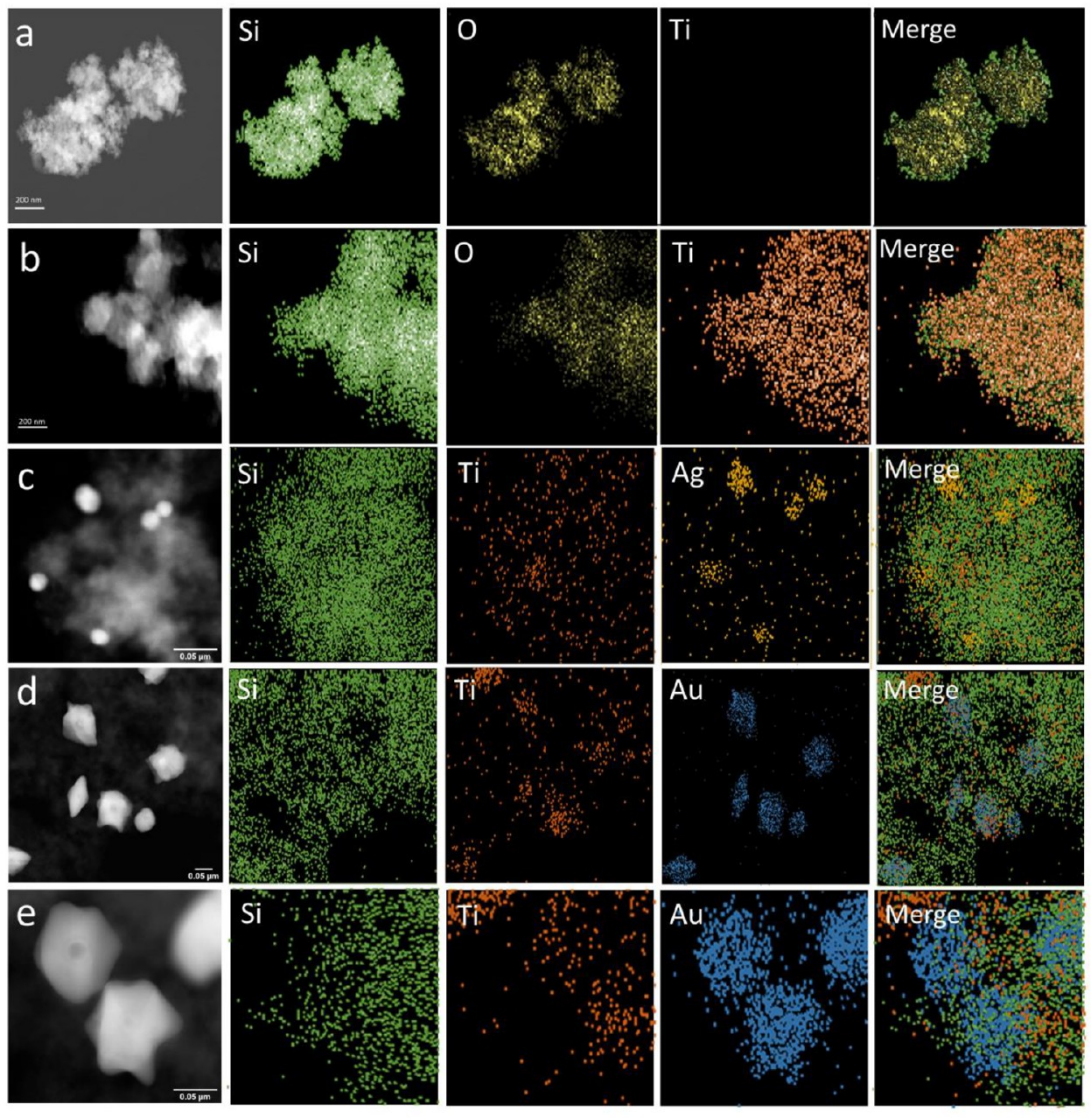
STEM images and elemental mapping of SiO_2_ aerogel (a), SiO_2_/TiO_2_ composite aerogel (b), SiO_2_/TiO_2_/Ag NPs (c), and SiO_2_/TiO_2_/Ag@Au NSs (d, e).

Further, Ag@Au NSs were synthesized onto SiO_2_/TiO_2_/Ag NPs via a seed-mediated growth method at room temperature (Figure S2b, schematic representation). In this process, first, gold cations (Au^3+^) were reduced to zerovalent gold atoms (Au^0^) by L-AA to be deposited on the surface of Ag seeds, which were already attached to the SiO_2_/TiO_2_ aerogel matrix. During the growth process of NS, the presence of Ag^+^ ions plays a crucial role in catalyzing the reaction through a galvanic displacement (GR) approach. This catalytic activity facilitates particle growth and concurrently stabilizes the NS morphology.^40^ The use of L-AA as a reducing agent in combination with AgNO_3_ as a codirect agent promotes the blocking of specific crystallographic planes and allowing for the reduction of Au^3+^ in the appropriated sites. CTAB is responsible for the anisotropic growth of Au on Ag seed surface via an oriented attachment mechanism. CTAB molecules induce the growth of asymmetrical fcc lattices by preferential capping of certain crystallographic planes of Ag seeds.^27^ Along with the seed-mediated growth process, the formation of Ag@Au NSs with an average final size of 97.5*/1.05 nm on an aerogel matrix was observed by TEM analysis (Figures 3d and S3b). XRD analysis was executed to assess the crystalline structure of the final composite aerogel (Figure 3e). A noticeable decrease was observed at the (101) peak of titanium followed by the growth of Ag NPs on the aerogel structure. This can be attributed to crystalline anatase TiO_2_ suppression as a result of the deposition of silver in the environment on TiO_2_.^41^ The pattern from SiO_2_/TiO_2_/Ag@Au NSs clearly shows that the characteristic peaks are assigned to the diffraction of (111), (200), (220), and (311) planes of Au corresponding to 2θ angles at 38.14°, 44.38°, 64.58°, and 77.66°. The obtained XRD pattern confirmed the face-centered cubic (fcc) crystal structure of Au (JDPDC file card no. 04–0784). The relatively higher peak formed at 38.14° showed that the preferential alignment of the (111) orientation was generated in Ag@Au NSs.^25,42^ STEM mapping analysis (Figure 4) showed that the main contribution of NSs is Au, which is also confirmed by the ICP-OES analysis (Table S3). The amount of Au in the final structure was found to be relatively high compared with Ag which can be clarified by the GR reactions between two metals driven by the oxidation and dealloying of Ag from the template, as well as the reduction and doping of Au to the structure. Additionally, the resulting Ag@Au NSs structures were characterized with a void in their body center which is the anticipated morphological variation throughout the GR process.^25^

Figure 3f represents diffuse reflectance spectra of SiO_2_ and SiO_2_/TiO_2_ aerogels and Tac plots of samples with their band gap calculations using the Kubelka–Munk function for indirect TiO_2_. Accordingly, by growing TiO_2_ within the SiO_2_ aerogel matrix, we successfully obtained anatase TiO_2_ with a lower band gap energy value (2.3 eV) and excitation threshold compared with traditional anatase TiO_2_ NPs with a 3.2 eV band gap energy value. The reduction in the energy band gap value can be correlated with the occurrence of lattice defects and oxygen vacancies^39^ and the SiO_2_–TiO_2_ heterojunction via the Ti–O–Si linkage during the synthesis. Ti–O–Si linkage can efficiently transfer the photoexcited electron, which can allow the structure to be photoactive for solar and visible light exposures.^36^ This result demonstrated that the TiO_2_ embedded into the aerogel matrix can assist as a much more effective semiconductor with improved photoactivity.

Figure S3a shows the color changes of the dispersion using anisotropic Ag@Au NSs formation on SiO_2_/TiO_2_ aerogels as a function of time. The blue/brownish color appeared in association with the growth of Ag@Au NSs stemming from their enhanced optical properties compared to spherical Ag NPs. Following the conversion of Ag NPs into Ag@Au NSs, a significant broad peak was formed in the vis/NIR region of the spectrum correlated with the LSPR of Ag@Au NSs (Figure 3g). The surface plasmon resonance of Ag@Au NSs allows these nanostructures to absorb strong light in the vis/NIR regions. Due to the large variation in the collective oscillations of the surface free electrons after Au addition, the clear shift to the NIR region of the spectra occurred following Ag@Au NSs formation. The peak position and intensity of the LSPR depend on the shape, size, and composition of the particles. In this manner, Ag@Au NSs with anisotropic morphology are responsible for the redshift of the plasmon. This redshift and broad absorption in visible range show that there is a large contribution of Au in the final nanostructure, which is also confirmed by color variations.

### 3.2 Photocatalysis Studies

Photocatalysis studies were first carried out by using the model organic dye, RhB. In this process, the photogenerated ROS react with RhB and break down dye structure into its smaller and harmless molecules.^43^ In general, the photodegradation efficiency differs depending on the types and levels of released ROS by the photocatalyst. Thus, the photocatalysis reactions in which RhB dye was used as a chemical probe were performed to investigate the ROS production capabilities of designed plasmonic-enriched composite aerogels and to make a preliminary prediction about their ROS-related phototoxicity against *E. coli*.

With the inspiration of the previous studies in which the photocatalytic activities of SiO_2_/TiO_2_ aerogels were evaluated,^15,36^ we investigated the effectiveness of our materials under solar and visible light exposures. Hence, SiO_2_ aerogels, commercial TiO_2_ NPs (anatase, 5 nm), and SiO_2_/TiO_2_ composite aerogel samples were mixed overnight with a RhB aqueous solution (10^–5^ M) to achieve an adsorption–desorption equilibrium between the particles and dye molecules. Following, the samples that adsorbed some of RhB dye were separated from the solution by centrifugation and the remaining RhB in the supernatant was determined by UV–vis spectroscopy. Accordingly, 26%, 9%, and 56% of RhB were adsorbed on pores/surface of SiO_2_, TiO_2_, and SiO_2_/TiO_2_ composite aerogels, respectively (Figure S4). The RhB dye adsorption capacities of SiO_2_ and SiO_2_/TiO_2_ composite aerogels were found higher than those of TiO_2_ NPs (9%) alone owing to their higher surface areas, porosities, and abundance of hydroxyl groups on the surface which can interact directly with RhB. Interestingly, SiO_2_/TiO_2_ composite aerogels showed the most efficient adsorption that can be explained by the stronger electrostatic interactions of cationic RhB dye with SiO_2_/TiO_2_ composite, which has a more negative surface potential compared to SiO_2_ (Figure S5).

**Figure 5:**
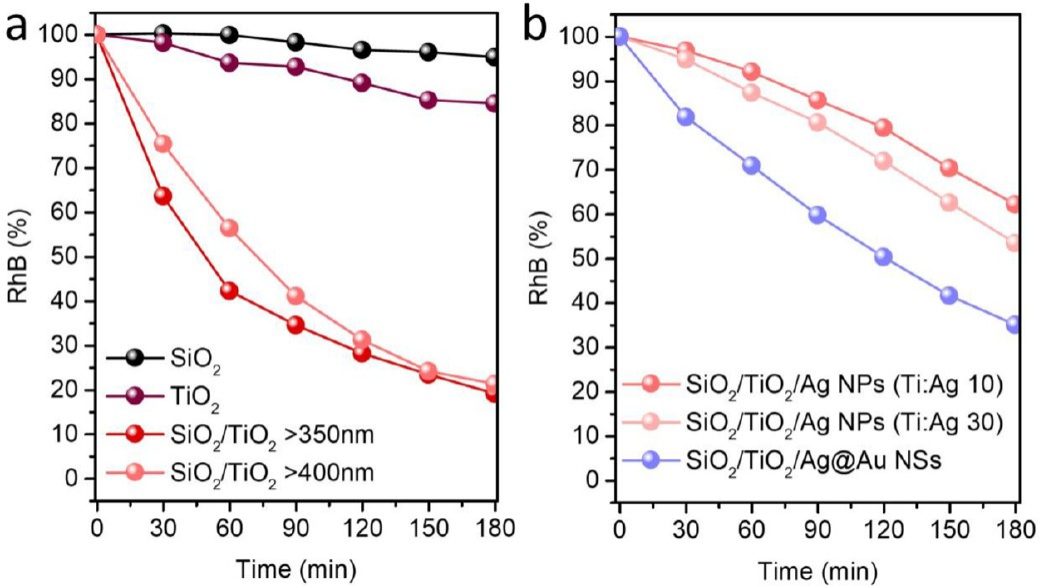
Photodegradation studies of RhB dye under solar (350–2400 nm) and visible light (400–2400 nm) irradiations in the presence of different SiO_2_/TiO_2_ catalysts. RhB photodegradation graphs for SiO_2_, TiO_2_, and SiO_2_/TiO_2_ samples under solar light exposure and SiO_2_/TiO_2_ sample under visible light exposure (a) for SiO_2_/TiO_2_/Ag NPs (Ti:Ag 10 and 30) and SiO_2_/TiO_2_/Ag@Au NSs under visible light exposure (b).

Afterward, the samples were illuminated by solar light (>350 nm of wavelength) for 3 h by keeping the temperature constant at 25 °C by water circulation. The photodegradation of RhB overtime in the presence of different aerogel samples was monitored by following the variations at its maximum absorbance (554 nm). The degradation percentages and shifts in the absorption spectra of RhB during the photodegradation process are shown in Figures 5 and S6. Since SiO_2_ aerogels are not photoactive materials, there was only 6% of RhB degradation over light irradiation (Figures 5a and S6a). Similarly, commercial TiO_2_ NPs showed very low photocatalytic activity with only 12% of RhB degradation after 3 h of reaction (Figures 5a and S6b). This result can be correlated with their UV-light limited photoactivity.^44^ Remarkably, SiO_2_/TiO_2_ composite aerogels showed highly efficient photoactivity by degrading 76% of RhB dye under solar light exposure in 3 h which is higher than TiO_2_ NPs (6.3-fold higher). The efficient photoactivity of anatase TiO_2_ in SiO_2_/TiO_2_ structures can be attributed to the presence of the SiO_2_ aerogel matrix, which ensures efficient adsorption sites for dye molecules and, consequently, enhanced interactions between TiO_2_ and RhB dye. Moreover, the abundance of hydroxyl groups in the aerogel matrix, the possible photogeneration of Ti^3+^ ions from titanium lattice defects, and the decreasing final energy band gap values (2.3 eV, in our case) were responsible for the increased ROS generation efficiency of SiO_2_/TiO_2_ composite aerogels.^17,45^ Besides, it is proved that the formation of oxygen vacancies and lattice defects (confirmed by XPS) during the composite aerogel synthesis enhances the photocatalytic activity by increasing the adsorption of organic compounds.^38^

To understand the photocatalysis mechanism behind the SiO_2_/TiO_2_ aerogel structures, aerogels were also irradiated by light with the wavelength of 400 nm cutoff range (>400 nm). Figure S6c,d represents the absorption spectra of RhB during the degradation process by SiO_2_/TiO_2_ under solar and visible light. In the case of the photocatalytic activity under the solar light exposure, the intensity of absorption spectra of RhB decreased and shifted to the maximum of 520 nm which indicated that N-de-ethylation and direct cleavage of chromophore ring processes coexisted under solar light irradiation. On the other hand, the photodegradation process went mainly through the N-de-ethylation process under visible light irradiation which mainly has a characteristic hypsochromic shift of maximum absorbance of RhB but no decrease in the maximum absorbance. The differences between two RhB degradation pathways under different light exposure conditions are directly related to the photogenerated radicals which are ^•^O_2_^–^ responsible for the N-de-ethylation processes and ^•^OH responsible for the direct cleavage.^46,47^ Thus, the radicals produced during the process were examined by using the well-known radical scavenger of TA to better understand the reason for the differences in RhB spectra. TA is a convenient chemical probe molecule that reacts with ^•^OH radicals in the medium and consequently transforms into fluorescent 2-hydroxy terephthalic acid (2-HTA) (Figure S7a).^48^ Accordingly, SiO_2_/TiO_2_ aerogels were dispersed in TA aqueous solution and illuminated with solar and visible light, separately. The conversion of TA into 2-HTA over time was followed by fluorescence spectroscopy. The obtained fluorescence spectra of 2-HTA in both cases are shown in Figure S7b. In the case of solar light, the fluorescence intensity of 2-HTA was increased over the reaction time of SiO_2_/TiO_2_ photoirradiation, which confirms that ^•^OH radicals were produced throughout this process. Nevertheless, the spectra of 2-HTA did not change during visible light irradiation of SiO_2_/TiO_2_ aerogels which confirmed that ^•^OH generation was suppressed and the process mainly took place through N-de-ethylation in the presence of ^•^O_2_^–^ radicals under this condition. Further, the degradation products of RhB irradiated under light sources with different wavelengths were examined by mass spectrometry (Figure S8). Accordingly, in both cases, the reaction started with N-de-ethylation and oxidation processes and continued with chromophore cleavage in the final step. The sample irradiated by solar light passed a path with more oxidation and cleavage after 5 h of reaction compared to visible light. Conversely, the process proceeded mostly via a N-de-ethylation process during the first hours of visible light irradiation which indicated that SiO_2_/TiO_2_ composites do not support sufficient ^•^OH production in the absence of UV-portion of electromagnetic spectrum.

Further, the photodegradation studies of RhB dye in the presence of SiO_2_/TiO_2_/Ag composite aerogels with different molar ratios of Ti:Ag (10 and 30) were performed under visible light irradiation (Figure 5b). Herein, we evaluated the plasmon-assisted photocatalytic activity of composite aerogels by changing the molar ratio of Ti:Ag which is an important parameter that directly regulate the efficiency of the hot-electron transfer mechanism.^41^ The excess amount of Ag NPs in the structure can increase their antibacterial activities; however, at the same time, it may adversely affect the dye degradation process because of the fast electron–hole recombination rates. Accordingly, during the photodegradation studies under visible light illumination of samples including different amounts of Ag NPs, RhB spectra were shifted and decreased in time, which clarified that the mechanism went through the N-de-ethylation and direct cleavage processes at the same time. Unlike the behavior of the sample containing only titanium under visible light, the sample containing Ag NPs promoted the formation of radicals (^•^OH and ^•^O_2_^–^, simultaneously) and, thus, can demonstrate that both mechanisms played simultaneous roles during dye degradation. Moreover, the photodegradation results showed that reducing the amount of Ag (Ti:Ag 30) resulted in increased RhB degradation ability, which confirmed that the importance of the ratio between Ti:Ag should be optimized to evade fast electron–hole recombination (Figures 5b, S6e, and S7b).

Finally, we performed the photocatalysis studies under visible light irradiation by using a Ag@Au NSs enriched SiO_2_/TiO_2_ aerogel matrix. As can be seen in the degradation plots and the RhB absorbance during visible light irradiation in the presence of photocatalysts (Figures 5b, S6f, and S7b), particles with Ag@Au NSs showed the highest photodegradation ability. The increased photocatalytic performance of Ag@Au NSs can be explained by two reasons: (i) the presence of Ag–Au bimetallic structure and (ii) the morphology of particles which enhanced the optical properties of plasmonic structures by means of their sharp tips. The growth of metallic Au onto Ag NPs ensures particle stability, and the presence of these two plasmonic components in the structure greatly improves their final optical properties. Also, the anisotropic morphology of the star-shaped structure causes an increase in the electromagnetic field at their tips. Hence, the ability of electron transfer to the valence band of TiO_2_ was boosted via photogenerated hot spots by means of a hot electron injection mechanism.

### 3.3 Phototoxicity Studies against *E. coli* Bacteria

**Figure 6:**
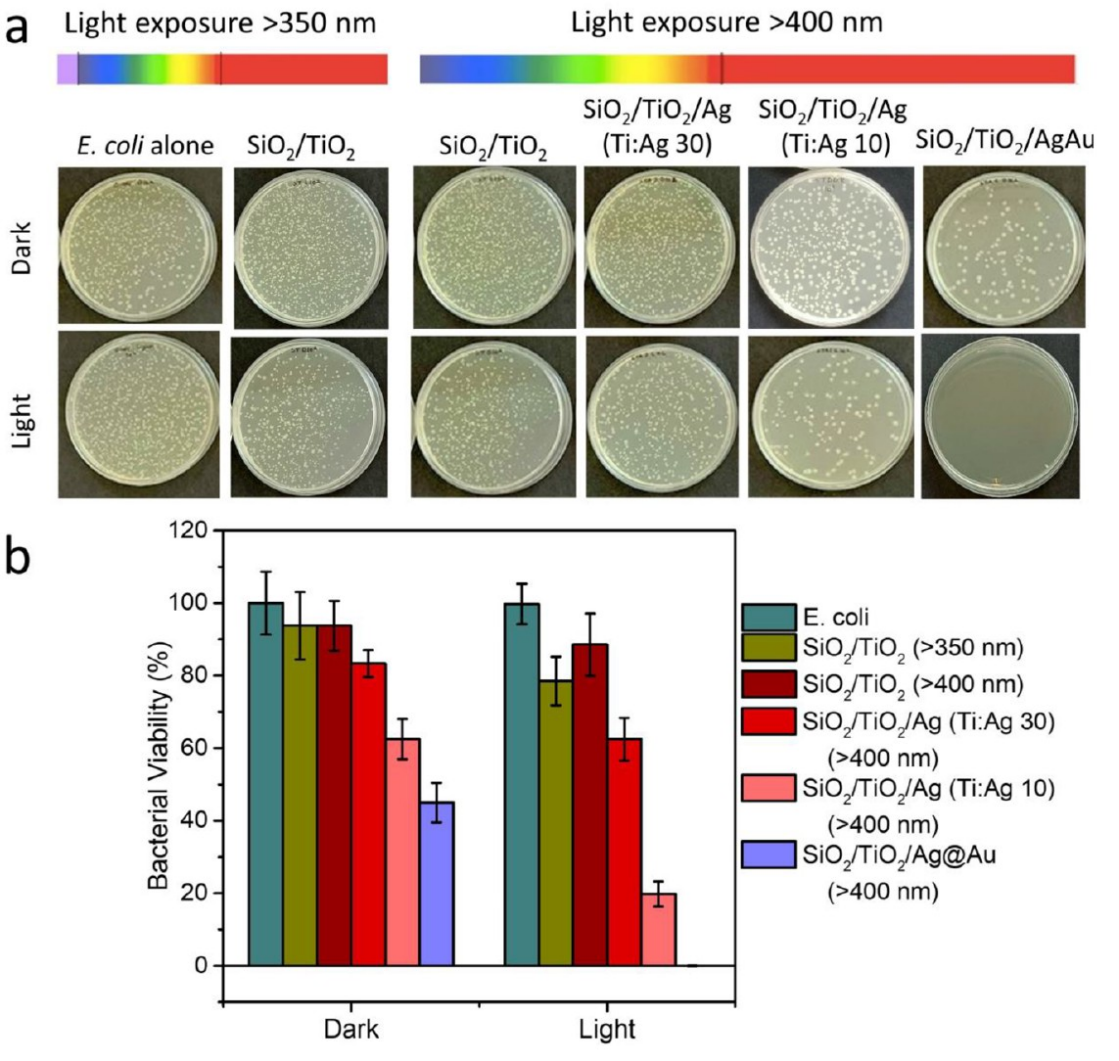
CFU assays of *E. coli* bacteria after exposure to solar and visible light in the presence of composite aerogels. The photographs of obtained nutrient agars after the phototoxicity experiments (a) and bacterial viability (%) graph calculated by counting *E. coli* colonies from these agars (b).

Photoinduced antibacterial effects of synthesized composite aerogels were investigated against *E. coli* bacteria, which is selected as the model strain. CFU count assay was performed to evaluate the biocidal activity of composites. Briefly, SiO_2_/TiO_2_, SiO_2_/TiO_2_/Ag NPs (Ti:Ag 10 and 30), and SiO_2_/TiO_2_/Ag@Au NSs samples with the concentration of 1 mg mL^–1^ were added in bacteria solution (in PSW), and the slurry was placed onto the stirrer to ensure continuous mixing during the experiments. Subsequently, bacteria-aerogel dispersions were irradiated for 3 h by solar (>350 nm wavelength) and visible (>400 nm wavelength) light or left in the dark for the control experiments. Phototoxicity tests were carried out in a water bath at 25 °C to prevent bacterial death caused by overheating during light exposure. After 3 h of irradiations, dispersions were serially diluted and seeded on nutrient agar plates. The bacterial agars were then incubated overnight at 37 °C to allow bacterial growth. Figure 6a,b represents all agars and bacterial viability percentages calculated by CFU assay after overnight incubation. As can be seen in the first column of agar plates and the viability bars, *E. coli* alone was resistant to light exposures. However, in the presence of SiO_2_/TiO_2_ composite aerogels, bacterial viability decreased to 93.7%, 78.5%, and 88.5% under dark, solar, and visible light conditions, respectively. Accordingly, SiO_2_/TiO_2_ aerogel structures did not show a significant effect against bacteria in the absence of light. This behavior was also confirmed by other reports in which TiO_2_ NPs have negligible toxicity to microorganisms even at concentrations greater than or equal to 100 ppm.^49^ On the contrary, it was assumed that TiO_2_ NPs irradiated by UV light could exert a significant bactericidal effect at 1 ppm concentration.^50,51^ Considering the conditions applied in this study, although a small portion of UV light (4%) caused a slight decrease in viability in solar light experiments, the absence of this portion in visible light experiments negatively affected the bactericidal activity. The insignificant decrease in bacterial viability under visible light exposure may be correlated to ^•^O_2_^–^ radicals produced, which we confirmed in the RhB photodegradation studies above. Nevertheless, these radicals were not sufficient to show effective antibacterial activity on *E. coli* and proved that ^•^OH radicals are also necessary to achieve an effective phototoxicity against this bacterial strain.

**Figure 7:**
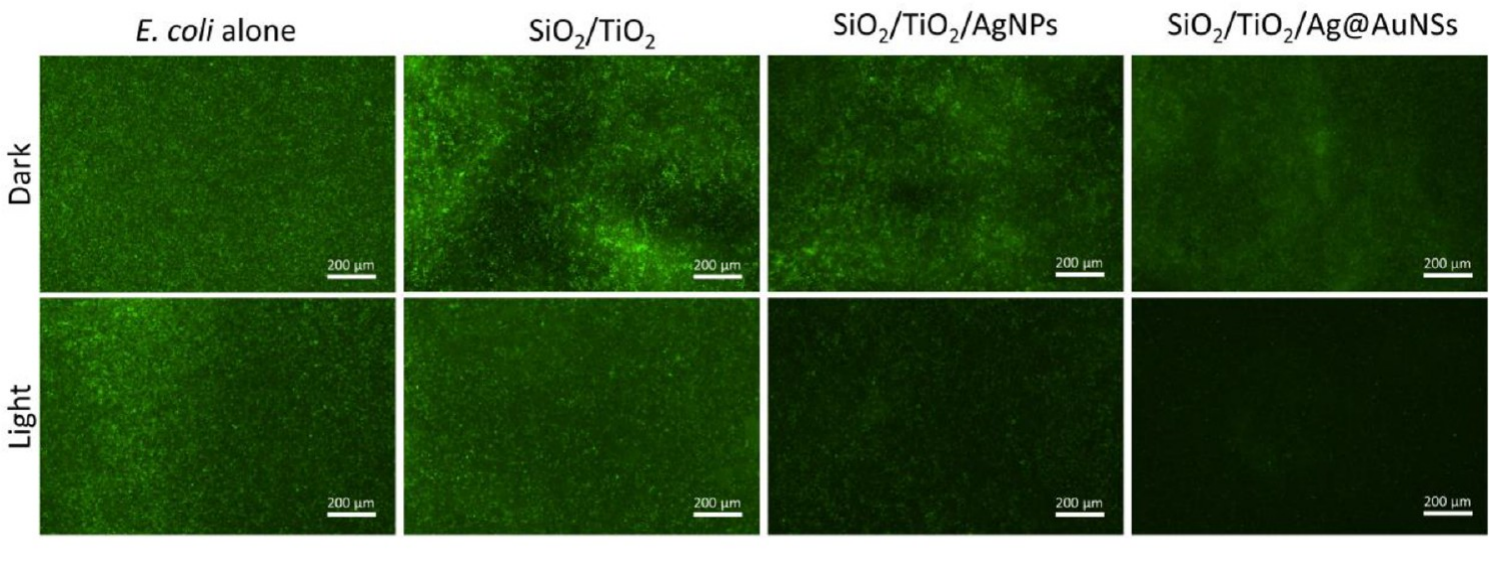
Fluorescence microscope images of *E. coli* bacteria expressing GFP grown overnight on SiO_2_/TiO_2_, SiO_2_/TiO_2_/AgNPs (Ti:Ag 10), and SiO_2_/TiO_2_/Ag@AuNSs composite aerogels (the images at first row, indicated as dark) and after the visible light irradiations for 3 h (the images at second row, indicated as light).

The phototoxicity against *E. coli* was further boosted by the addition of Ag NPs onto the SiO_2_/TiO_2_ aerogel matrix, especially when we increased the concentration of Ag NPs on the structures (Figure 6). Increasing the amount of Ag NPs under dark conditions in both samples (Ti:Ag 30 and Ti:Ag 10) resulted in a reduction in *E. coli* viability, simultaneously. These results proved that the main contribution to the toxicity against *E. coli* bacteria in the absence of light was the released Ag^+^ ions themselves (83.3% in the case of Ti:Ag 30 and 62.5% in the case of Ti:Ag 10). Besides, as a result of visible light irradiation experiments, the antibacterial activity was greatly increased, where we experienced a great deal of the effect of ^•^OH and ^•^O_2_^–^ radicals formed as a result of the plasmon-assisted photoactivity in addition with the released Ag^+^ ions toxicity. Importantly, although the increase in the amount of Ag NPs on the composite aerogel matrix adversely affected the production of ROS (Figure 5b and Figure S6), it was observed that high concentrations of Ag NPs were much more effective in boosting the toxicity (62.5% and 19.8% of bacterial viabilities in the case of Ti:Ag 30 and Ti:Ag 10, respectively). This can be attributed to the synergistic effect resulting from the simultaneous increased Ag^+^ ion release and ROS generation. However, some studies showed during light irradiation that the released Ag^+^ ions can be reduced to Ag^0^ by photon energy to deposit back into their NP forms. Hereby, Ag^+^ ion concentrations are almost negligible for bacteria death in the photocatalyst system, which provides other mechanisms for the inactivation of bacteria.^23^ Accordingly, we hypothesized that the greatest contribution to antibacterial activity under visible light exposure was related to ROS formation. To prove that, we performed the antibacterial experiments under the same conditions but using SiO_2_/TiO_2_/Ag@Au NSs structures. Consequently, the samples decorated with Ag@Au NSs showed the most effective toxicity by giving the viability percentages of bacteria 31.25% under the dark and 0% under the visible light irradiations for 3 h (Figure 6). Accordingly, the potent toxicity of composite aerogels decorated with Ag@Au NSs against bacteria under dark conditions can be attributed to the metal content (now highly low amounts of Ag but excess amounts of Au, see Table S3) and most likely the anisotropic morphology of Ag@Au NSs with sharp tips. The anisotropic morphology of Ag@Au NSs provides higher surface area and consequently the active adsorption sites for bacteria that can easily attach via interactions facilitated by sharp tips of plasmonic NSs. Lastly, as can be seen in Figure 6 in the last column, the phototoxicity of these particles under visible light exposure for 3 h killed all of the bacteria with superior activity compared with other samples. Associated with their improved optical properties (Figure 3g, UV–vis spectra), bimetallic nature, and photocatalytic activity on the organic dyes, Ag@Au NSs exhibited improved ROS generation under visible light and consequently the effective phototoxicity on *E. coli* bacteria. Moreover, the obtained results were confirmed by fluorescence microscopy (Figure 7). Herein, bacterial viability was investigated by monitoring the fluorescent green color given by bacteria in relation to their GFP expression. Accordingly, in parallel with the CFU results, it was observed that the bacterial viability decreased significantly, especially in the environment with Ag@Au NSs.

## 4 Conclusions

SiO_2_ aerogels with high surface areas, porosities, and thermal stabilities are remarkable materials as photocatalyst support. Herein, TiO_2_ NPs, well-known semiconductor materials, were grown on a SiO_2_ aerogel matrix to enhance their photoactivity activity. Therefore, SiO_2_ aerogels ensured the homogeneous distribution of TiO_2_ NPs, and at the same time, effective adsorption sites for organic molecules during photodegradation reactions were provided. Thus, the loss of efficiency caused by particle aggregation was eliminated, and the efficiency was significantly increased by ensuring that the adsorbed molecules into the aerogel pores come into close contact with TiO_2_ NPs. Furthermore, Ag NPs were synthesized *in situ* on this matrix, and their photocatalytic activities were investigated in detail using the organic dye RhB. Independently of Ag NPs, SiO_2_/TiO_2_ composite aerogels showed high dye degradation profiles under solar light irradiation owing to their improved final properties. Although the presence of Ag NPs is important for antibacterial activity, the use of excess amounts of Ag NPs reduced photocatalytic activity which can be related to the clogged pores by Ag NPs and thereby the reduced surface area of final aerogel matrix and the fast electron–hole recombination rates between TiO_2_ and excess amounts of Ag NPs. In addition, the rapid oxidation and low stability of Ag NPs considerably shorten the life of the material. In order to increase the stability of the plasmonic structure and create a strong LSPR effect in the vis/NIR region during photocatalysis with titanium, for the first time anisotropic Ag@Au NSs were grown *in situ* on these composite aerogels using Ag NPs as nucleation centers. Thus, by plasmon-assisted photocatalysis reactions, effective ROS production was achieved under light irradiation in the visible region, where the photoactivity activities of TiO_2_ NPs are limited. It should be noted that although the current design in this study did not exhibit high RhB degradation percentages, it can be improved by optimizing plasmonic metal concentrations in the aerogel matrix. Herein, attention has been paid to optimizing the contributions of ROS formation and possible metal ion release (based on the Ag NPs amounts), as the aim is to ensure the most effective system for microbial disinfection. After evaluating the photoreactivity of the designed aerogel system on RhB organic dye, its phototoxic effects against *E. coli* bacterial strain were investigated. Our design showed that plasmon-enriched composite aerogels efficiently boosted the ROS production under visible light exposures and that the structures containing Ag@Au NSs showed a much more effective antibacterial effect compared to their counterparts.

## Supporting Information Available

Table of the specific surface area and pore volume/size values provided from N_2_ adsorption/desorption analysis, table of surface atomic percentages maintained from XPS analysis, and table of concentrations of elements analyzed by ICP-OES, XPS survey spectra of SiO_2_ and SiO_2_/TiO_2_ composite aerogels, schematic representation of the growth mechanism of Ag NPs and Ag@Au NSs structures, photographs of the color change of growth solution during seed-mediated synthesis of Ag@Au NSs on SiO_2_/TiO_2_/Ag NPs aerogel matrix, size distribution graphs of Ag NPs and Ag@Au NSs grown on SiO_2_/TiO_2_ aerogel matrix, RhB adsorption studies, zeta potential values, UV–vis spectra of the RhB dye during photoirradiation studies, the conversion of TA to fluorescent 2-HTA molecules through the reaction with ^•^OH radicals, fluorescence spectra of 2-HTA generated during photoirradiation of composite aerogels under solar and visible light irradiations, and mass spectroscopy analysis of RhB products after the photodegradation studies (PDF)
