# Supplementary material for: Plasmon-Sensitized Silica-Titanium Aerogels as Potential Photocatalysts for Organic Pollutants and Bacterial Strains
Source: ACS Omega. 2023 Sep 4;8(37):33857–69. doi: 10.1021/acsomega.3c04556 (PMC10515350; doi:10.1021/acsomega.3c04556)
Supplement: Supplementary file 1 — ao3c04556_si_001.pdf [file ao3c04556_si_001.pdf]

Supporting Information

Plasmon-Sensitized Silica-Titanium Aerogels as Potential Photocatalysts for Organic Pollutants and Bacterial Strains

Ecem Tiryaki[1,2,*], *Sevil Yücel*[2] , *Miguel A. Correa-Duarte*[3]

[1]Nanomaterials for Biomedical Applications, Italian Institute of Technology (IIT), 16163, Genova, Italy

[2]Department of Bioengineering, Faculty of Chemical and Metallurgical Engineering, Yildiz Technical University, 34220, Esenler, Istanbul, Turkey

[3]CINBIO, Universidade Vigo, 36310 Vigo, Spain, Southern Galicia Institute of Health Research (IISGS), and CIBERSAM, 36310, Vigo, Spain

Table S1. The specific surface area and pore volume/size values of SiO_2 aerogels and SiO_2/TiO_2 composite aerogels with different titanium concentrations

	BET surface area (m^2g^{-1})	***BJH Desorption Pore volume (cm^3g^{-1})***	***BJH Desorption Pore size (nm)***
SiO_2	447.9	0.7	8.1
SiO_2-TiO_2 (0.04M)	379.8	0.6	8.5
SiO_2-TiO_2 (0.09M)	282.8	0.4	8.3
SiO_2-TiO_2 (0.17M)	178.8	0.3	7.9

Table S2. Surface atomic percentages of main elements in SiO_2 and SiO_2/TiO_2 aerogels (XPS analysis)

Material	***Atomic Percentage (at. %)***			
	Si2p	***Ti2p (Ti4+/Ti3+)***	***O1s***	***C1s***
SiO_2	32.5	-	64.9	2.4
SiO_2/TiO_2	29.6	2.0	63.6	3.9

Table S3. ICP-OES analysis of composite aerogel structures

Elements (mg L^{-1})	***SiO_2***	***SiO_2/TiO_2***	***SiO_2/TiO_2/Ag (Ti:Ag 10)***	***SiO_2/TiO_2/Ag (Ti:Ag 30)***	***SiO_2/TiO_2/Ag@Au***
Si	1309.0	689.0	557.0	470.0	309.0
Ti	<0.5	101.0	88.0	102.3	50.9
Ag	<0.5	<0.5	7.9	3.2	1.8
Au	<0.5	<0.5	<0.5	<0.5	48.6

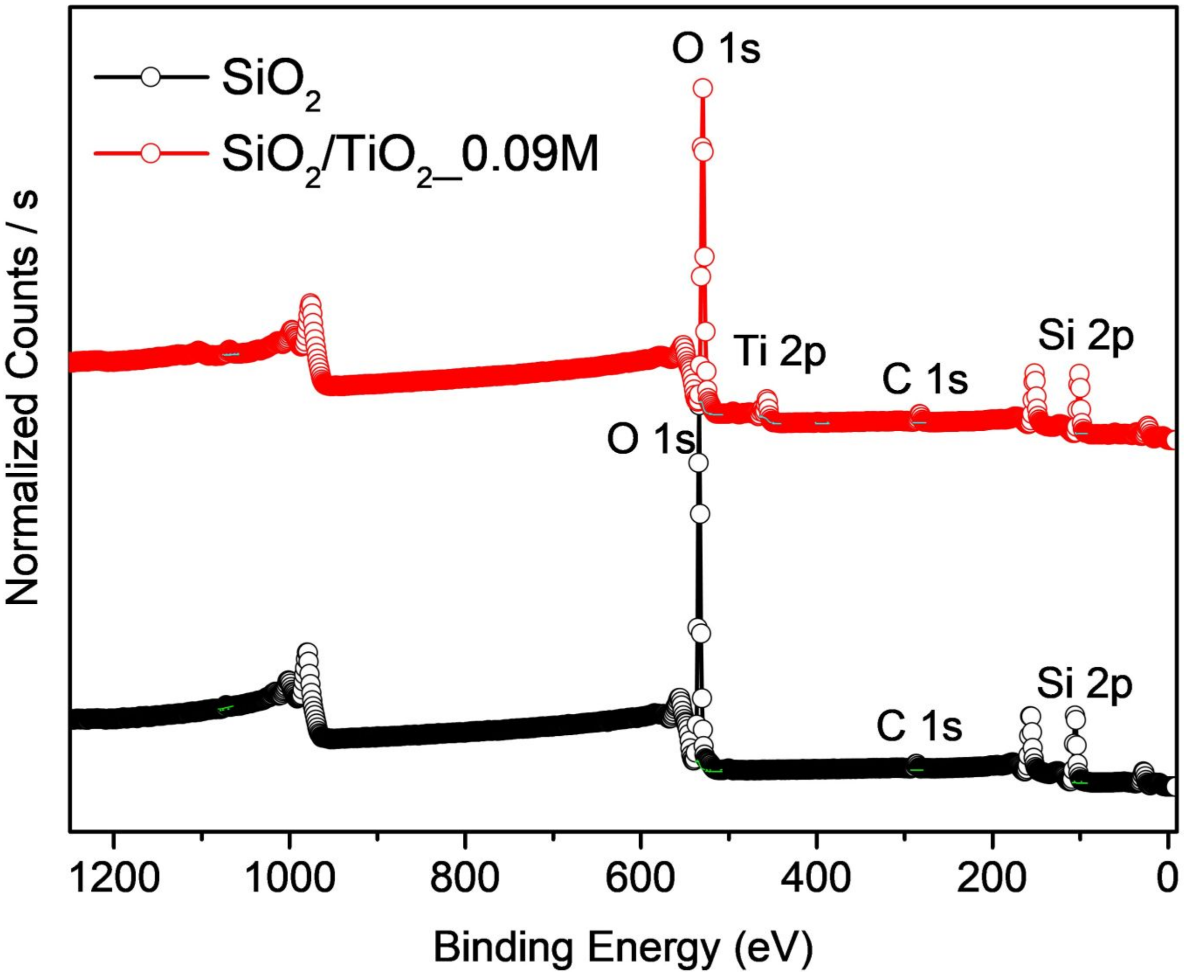

Figure S1. XPS survey spectra of SiO_2 and SiO_2/TiO_2 composite aerogels.

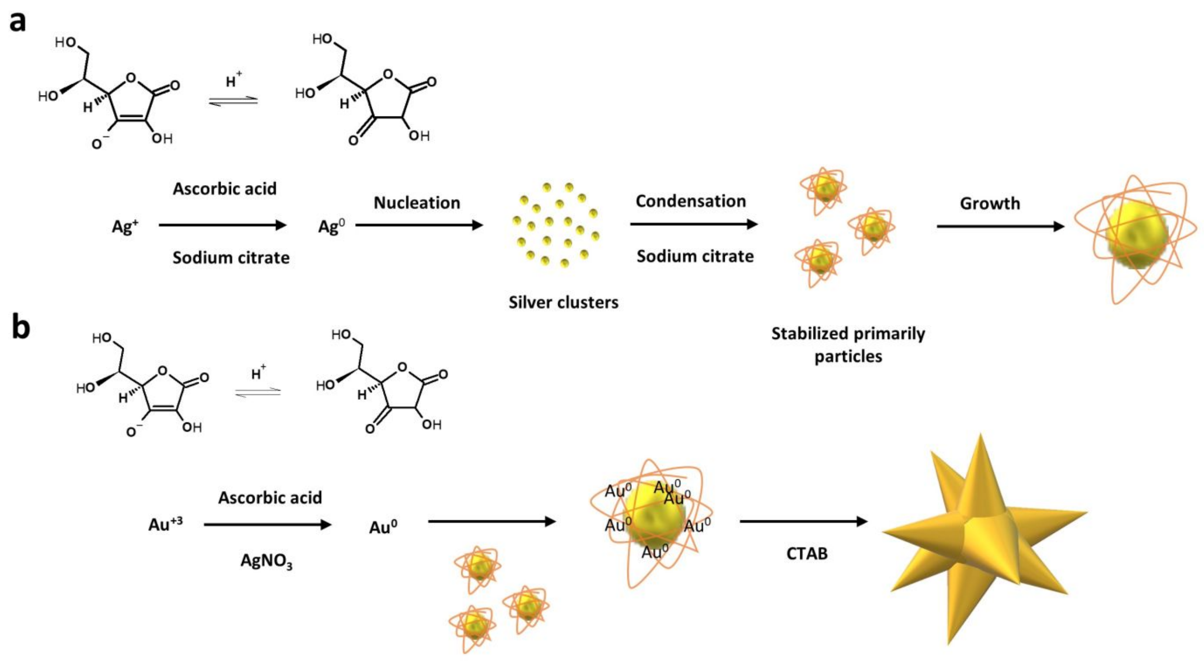

Figure S2. Schematic representation of the synthesis of Ag NPs via chemical reduction method (a) and Ag@Au NSs via galvanic replacement method by using pre-synthesized Ag NPs as nucleation centers (b).

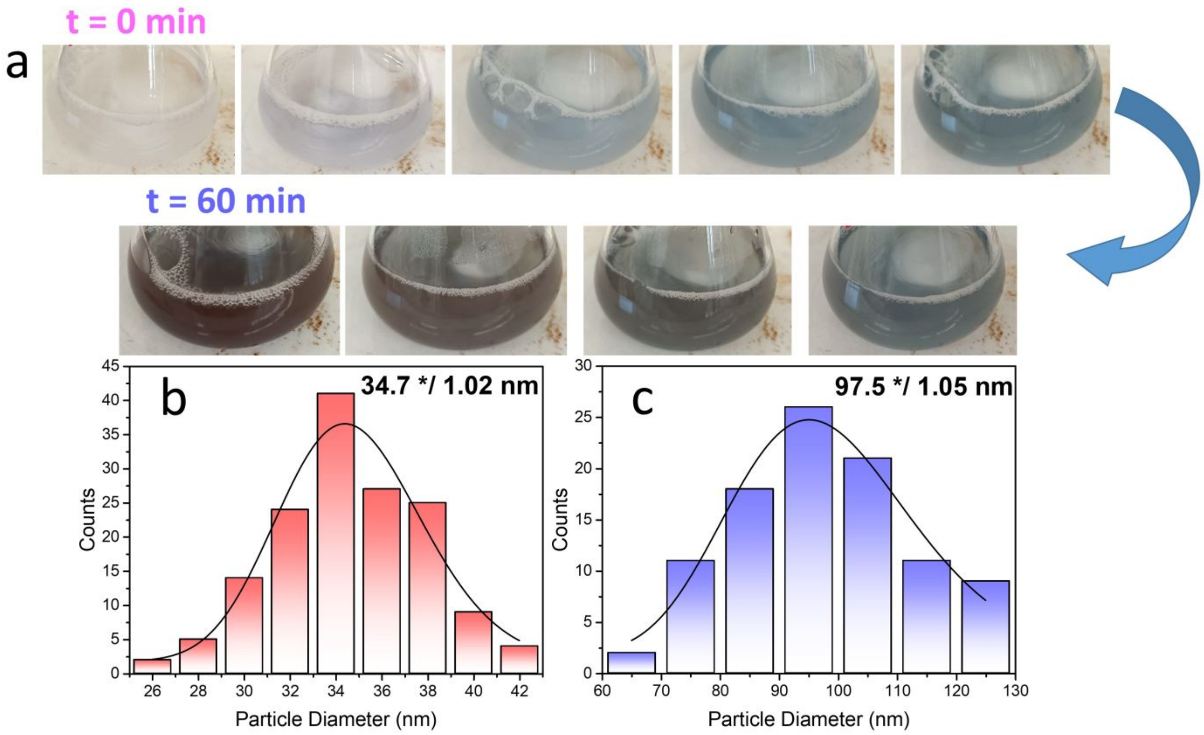

Figure S3. The color change of growth solution during the seed-mediated synthesis of Ag@Au NSs onto SiO_2/TiO_2/Ag NPs aerogel matrix (a), size distribution graphs of Ag NPs (b) and Ag@Au NSs (tip to tip) (c) grown on SiO_2/TiO_2 aerogel matrix. The corresponding size distributions of particles were assigned using image analysis software ImageJ and fitting to a log-normal function, expressing in terms of the geometric mean and standard deviation as x^*/σ^2 (i.e., for an interval of confidence of 95.5%).

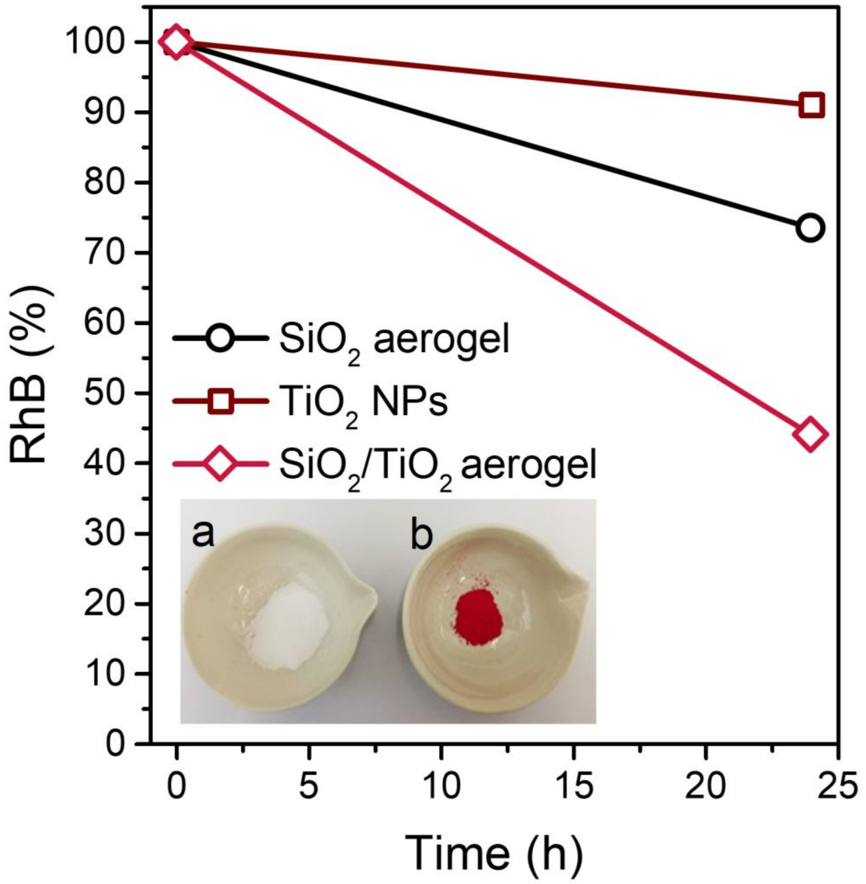

Figure S4. RhB adsorption percentages of different particles, (inset: the photograph of SiO_2/TiO_2 aerogels before (a) and after (b) RhB adsorption)

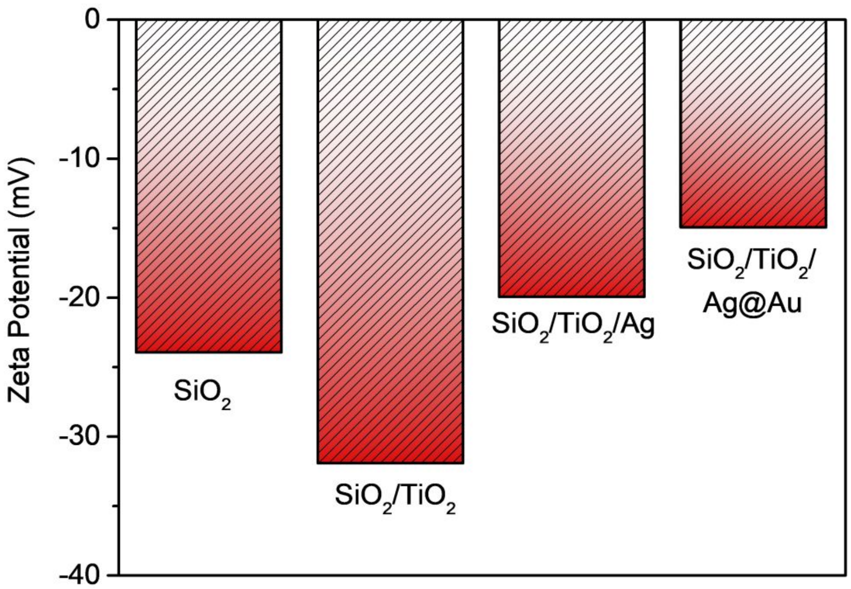

Figure S5. Zeta potential values of composite aerogels

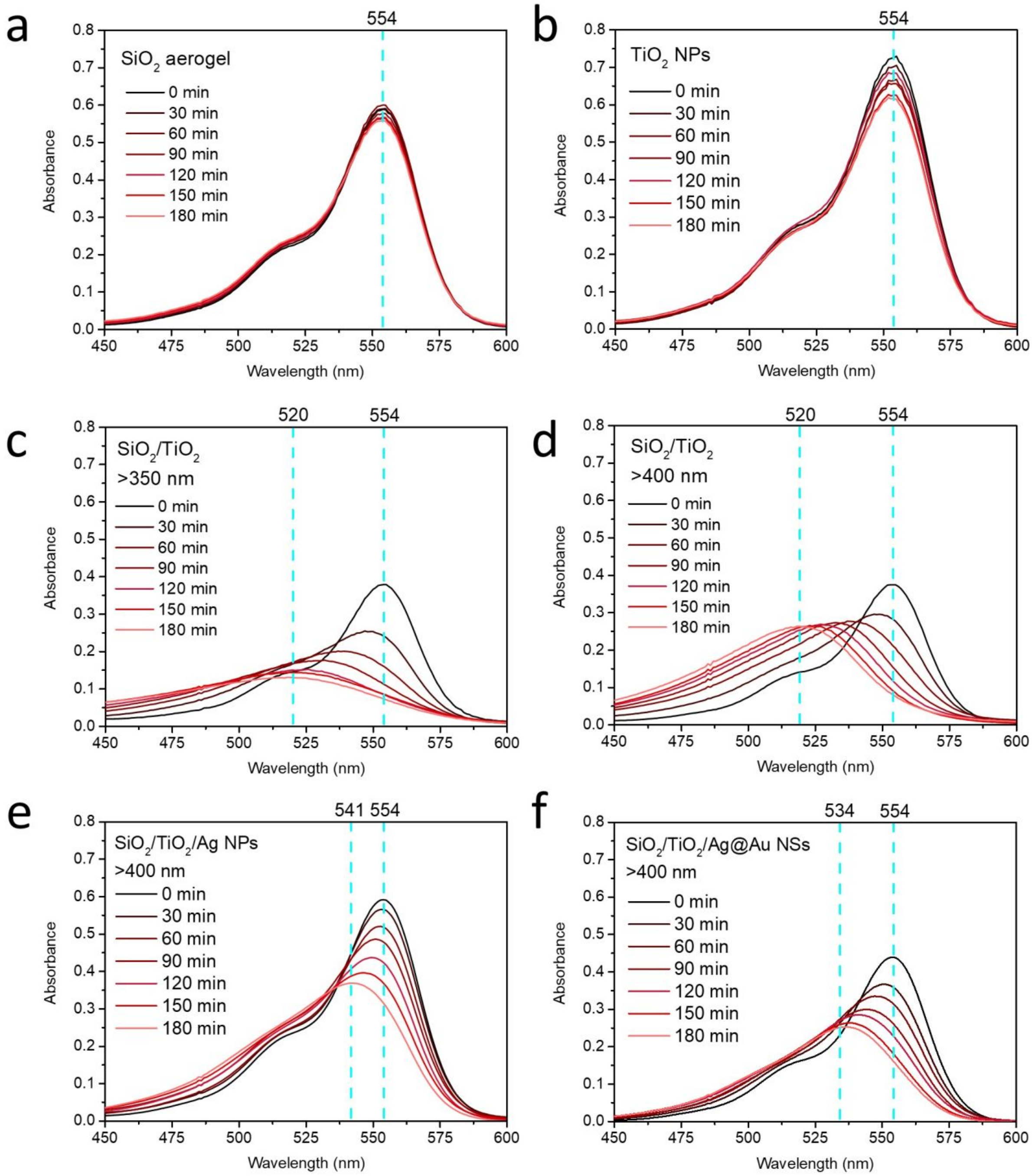

Figure S6. UV-vis spectra of RhB dye with the absorbance changes over time as a result of its photodegradation in the presence of SiO_2 aerogels (a), TiO_2 NPs (b), and SiO_2/TiO_2 aerogel (c) under solar-light irradiation (>350 nm), and SiO_2/TiO_2 aerogel (d), SiO_2/TiO_2/Ag NPs (Ti:Ag 30) (e), and SiO_2/TiO_2/Ag@Au NSs under visible-light irradiation (>400 nm) (f).

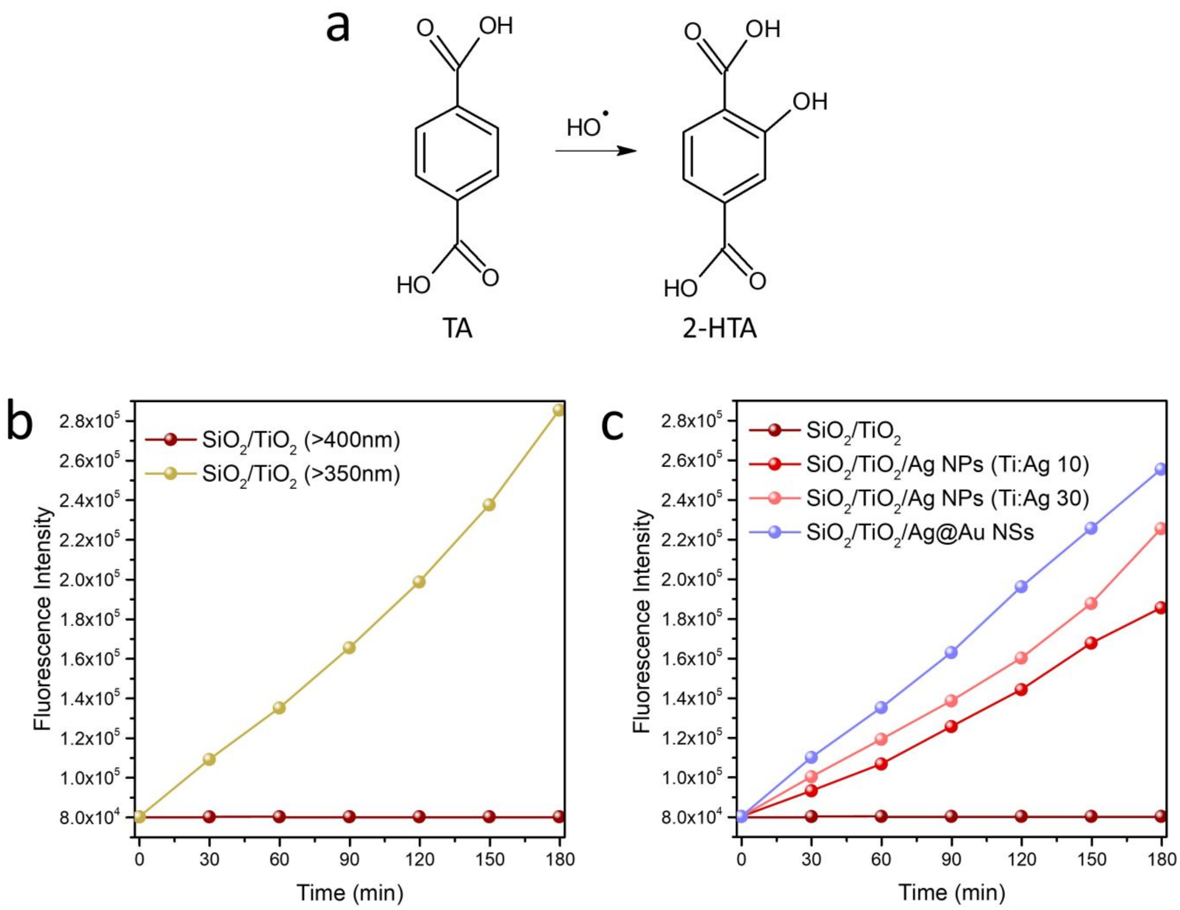

Figure S7. The conversion of TA to fluorescent 2-HTA molecules through the reaction with •OH radicals (a), Fluorescence spectra of 2-HTA generated by the reactions between TA and •OH radicals during photoirradiation of composite aerogels under solar and visible light irradiations. The spectra obtained from SiO_2/TiO_2 composite aerogels under solar (>350 nm)- and visible (>400 nm)-light irradiations (b), and from plasmonic NPs-enriched SiO_2/TiO_2 aerogels under visible (>400 nm)-light irradiation (c).

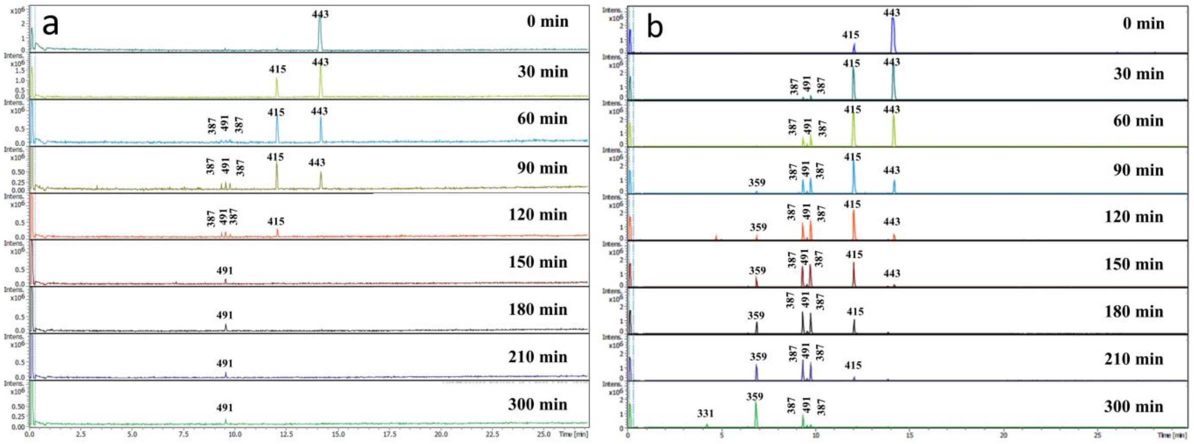

Figure S8. Mass spectroscopy analysis of RhB products after photodegradation studies under solar (a) and visible light (b) irradiations in the presence of SiO_2/TiO_2 aerogels
